# Recent Developments in Gene Therapy for Neovascular Age-Related Macular Degeneration: A Review

**DOI:** 10.3390/biomedicines11123221

**Published:** 2023-12-05

**Authors:** Lucia Finocchio, Marco Zeppieri, Andrea Gabai, Giacomo Toneatto, Leopoldo Spadea, Carlo Salati

**Affiliations:** 1 Department of Ophthalmology, University Hospital of Udine, 33100 Udine, Italy; 2 Eye Clinic, Policlinico Umberto I, “Sapienza” University of Rome, 00142 Rome, Italy

**Keywords:** gene therapy, neovascular AMD, clinical trials, maculopathy, target therapy

## Abstract

Age-related macular degeneration (AMD) is a complex and multifactorial disease and a leading cause of irreversible blindness in the elderly population. The anti-vascular endothelial growth factor (anti-VEGF) therapy has revolutionized the management and prognosis of neovascular AMD (nAMD) and is currently the standard of care for this disease. However, patients are required to receive repeated injections, imposing substantial social and economic burdens. The implementation of gene therapy methods to achieve sustained delivery of various therapeutic proteins holds the promise of a single treatment that could ameliorate the treatment challenges associated with chronic intravitreal therapy, and potentially improve visual outcomes. Several early-phase trials are currently underway, evaluating the safety and efficacy of gene therapy for nAMD; however, areas of controversy persist, including the therapeutic target, route of administration, and potential safety issues. In this review, we assess the evolution of gene therapy for nAMD and summarize several preclinical and early-stage clinical trials, exploring challenges and future directions.

## 1. Introduction

### 1.1. Definition of Age-Related Macular Degeneration

Age-related macular degeneration (AMD) is a chronic inflammatory eye disease that involves the macular region with a strong hereditary component, typically affecting people over 60 years of age. Pathologic changes involve the deeper retinal layers of the macula and surrounding vasculature, resulting in central vision loss. The disease has a prevalence of 8.7% worldwide [1] and is the most common cause of severe visual impairment in developed countries [2,3,4,5,6]. Its prevalence is likely expected to rise due to the exponential aging of the population. Principal risk factors for developing AMD include age, smoking history, hyperlipidemia, family history, and ethnicity [7]. There are two main types of AMD: non-neovascular and neovascular. Non-neovascular AMD (“dry” AMD) accounts for almost 80–85% of all cases and is usually related to a more favorable visual prognosis. The accumulation of retinal deposits, called drusen, is a distinctive clinical finding and may be the first sign of the “dry” form of the disease. Retinal pigment epithelial (RPE) changes, pigment clumping, or autofluorescence abnormalities may also be early clinical signs [8]. The type and quantity of drusen define the early, intermediate, and late stages of initial dry AMD but are not necessarily associated with vision impairment [9]. Progression to geographic atrophy (GA) and neovascular AMD (“wet” AMD or nAMD) is the key reason for severe vision loss as a result of AMD [10]. Neovascular AMD affects the remaining 15–20% of eyes with AMD and is characterized by the formation of macular neovascularization (MNV). These new blood vessels may cause an accumulation of subretinal, intraretinal, and sub-RPE fluid and bleeding, with metamorphopsia and central scotoma, respectively. Vascular endothelial growth factor (VEGF) is the main factor responsible for this abnormal vascular proliferation.

### 1.2. Pathophysiology of nAMD

Although there has been extensive research conducted to understand AMD pathogenesis, it remains not completely clear due to its multifactorial character. AMD is the consequence of the interaction between metabolism, genetics, and the environment.

The RPE promotes a vascular background along its basal surface and an avascular background along its apical surface. This creates an essential environment to maintain retinal photoreceptor cells in healthy conditions. Vascular endothelial cells require survival factors such as vascular endothelial growth factor (VEGF), basic fibroblast growth factor (bFGF), and angiopoietin-1 [11] that can originate from the extracellular matrix, surrounding cells, or plasma; VEGF represents an important mediator of paracrine and endocrine trophic support [12,13]. With aging, several changes happen in the RPE/Bruch’s membrane complex, modifying its capacity to remove residual substances such as lipofuscin [14], including the thickening of Bruch’s membrane, reduction of metabolic activity, loss of mitochondria and reduced choroidal blood supply [15]. Moreover, aging increases the risk of retinal and choroidal hypoxia. With age, the accumulation of lipofuscin, reactive oxygen species (ROS) and other factors cause a thickening of Bruch’s membrane, that not only decreases the removal of debris by the choriocapillaris but also acts as a barrier to the diffusion of oxygen and nutrients from the choroid to the photoreceptors and the RPE [16,17]. Hypoxia in turn causes an upregulation of a heterodimer made up of HIF-1α and HIF-1β, which is the Hypoxia-inducible factor-1 (HIF-1), [18]. HIF-1 regulates the transcription of genes of VEGF and its receptor VEGFR, platelet-derived growth factor-B (PDGF-B) and its receptor PDGFRβ, angiopoietin-2 (Ang2), stromal-derived factor-1 (SDF-1) and its receptor CXCR4, and vascular endothelial-protein tyrosine phosphatase (VE-PTP) [19]. HIF-1, VEGF, and VEGFR family are the principal intermediaries of angiogenesis regulation [20]. VEGF is the most studied factor in the context of ocular neovascularization. Encoded by the VEGF gene, this glycoprotein family, including VEGF-A, VEGF-B, VEGF-C, VEGF-D, VEGF-E, and VEGF-F and placental growth factor (PLGF), primarily activates cellular signaling pathways to facilitate the development of new blood vessels, either de novo or from existing ones. VEGF-A plays a crucial role in vascular proliferation and the migration of endothelial cells for both physiological and pathological angiogenesis [21,22]. However, VEGF expression by RPE cells seems to not be sufficient to cause MNV. In addition to secreted factors, which are mainly governed by HIF-1, signals contributing to MNV formation also encompass cues from the extracellular matrix and neighboring cells [12].

Inflammation is believed to play a central role in the pathogenesis of both dry and wet AMD. In the literature, there is strong evidence to suggest that an abnormal complement activation is significantly involved in the pathogenesis of the disease.

Even though complement activation products were detected in drusen nearly thirty years ago [23,24], it was not until the early 21st century that a major heritable determinant of AMD was identified as a single nucleotide polymorphism in the complement factor H (CFH) gene [25,26,27,28,29]. This discovery motivated genotyping initiatives in AMD, leading to the identification of further risk variants in other complement gene loci, such as C3, C2, CFB, C9, CFI, and CFHR4 [26,27,28,29,30,31,32,33,34]. C3a and C5a have been found in soft drusen and induce the upregulation of VEGF in RPE, increasing the risk of MNV associated with soft drusen [35].

The membrane attack complex (C5b-9, MAC) is also found in drusen and in impaired RPE cells of AMD-affected eyes [36]. Various factors are suggested to trigger complement activation in the retina, including oxidative stress and the buildup of pro-inflammatory derivatives from the visual cycle, such as lipofuscin components, apolipoproteins, and amyloid beta [37,38]. These elements are believed to interact with and amplify other established pathological mechanisms, like RPE dysfunction stemming from choroidal vascular insufficiency. Oxidative stress has the potential to disrupt the regulation of the complement system by RPE cells. This disruption includes a decrease in the surface expression of complement inhibitors, decay accelerating factor (CD55), and CD59, as well an impairment of complement regulation at the cell surface by CFH [39]. Furthermore, oxidative stress may impede interferon-γ from effectively enhancing CFH expression in RPE cells. In vitro, products resulting from the photo-oxidation of N-retinylidene-N-retinylethanolamine (A2E) in RPE can activate the complement system [40]. Choroidal dendritic cells and retinal microglia cells, both fundamental to retinal structure and metabolism, are “activated and recruited” by locally injured and/or sublethal damaged RPE cells, related to RPE blebs, fragments, and debris. They can maintain and enhance the local inflammation, not only activating the complement, but also forming an immune complex and recruiting choroidal T-cells or phagocytic cells, collectively contributing to the development of AMD [41,42]. Under the stimulation of inflammatory mediators, RPE cells produce cytokines and chemokines, including IL-4, -5, -6, -8, -10, -13, -17, IFN-β, IFN-γ, TGF-β, MCP-1, and VEGF. Inflammatory cytokines can also enhance the secretion of VEGF [43,44,45].

**Figure 1:**
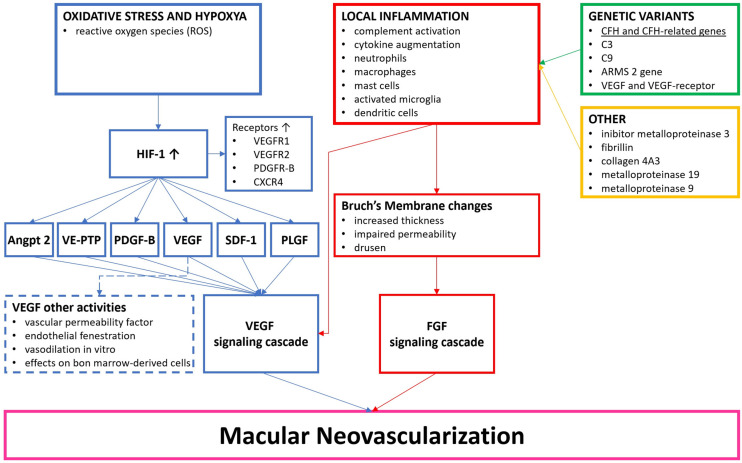
Overview of the pathophysiological process generating macular neovascularization (MNV). Adapted from Campochiaro, Anderson et al., Ricci et al. [12,36,45].

As discussed above, genetics plays a role in the pathogenesis of nAMD. Genetic variants implicated in nAMD encompass CFH, CFH-related genes, complement proteins C3 and C9, the age-related maculopathy susceptibility (ARMS)2 gene, and the VEGF and VEGFR axis [46,47,48,49,50,51,52,53]. Furthermore, inhibitor metalloproteinase (TIMP) 3, fibrillin, collagen 4A3, and metalloproteinase 19 and 9 appear to play a role in the development of nAMD [49]. (Figure 1).

### 1.3. Angiogenesis and VEGF Pathway

VEGF induces a vigorous angiogenic response in a variety of in vivo models [54,55]. VEGFR 1 and 2 are expressed in the cell surface of most blood endothelial cells. VEGFR-3, instead, is largely restricted to lymphatic endothelial cells. The primary contributor to angiogenesis is suggested to be VEGF-A, as it interacts with both VEGFR-1 and VEGFR-2 [56]. In contrast, PLGF and VEGF-B interact only with VEGFR-1; VEGF-E (ort-virus-derived) is a selective VEGFR-2 agonist; VEGF-C and VEGF-D bind VEGFR-2 and VEGFR-3. There is much evidence that VEGFR-2 is the major mediator of endothelial cell mitogenesis and survival, as well as angiogenesis and microvascular permeability [21]. In contrast, VEGFR-1 does not mediate an effective mitogenic signal in endothelial cells, and it may perform an inhibitory role by sequestering and preventing VEGF interaction with VEGFR-2 [57]. However, VEGFR-1 has an established signaling role in mediating monocyte chemotaxis [58].

## 2. Current Treatment Landscape in nAMD

### 2.1. Anti-VEGF Therapy: The Gold Standard

Intravitreal anti-VEGF therapies that target vascular permeability, angiogenesis, and inflammatory responses by inhibiting VEGF signaling are the current gold standard treatments for patients with nAMD [59]. Intravitreal anti-VEGF agents include aflibercept (Eylea^®^; Regeneron Pharmaceutical, Inc., Tarrytown, USA and Bayer Healthcare, Berlin, Germany) [60], ranibizumab (Lucentis^®^, Genentech, South San Francisco, CA, USA/Roche, Basel, Switzerland) [57,61] and bevacizumab (Avastin; Genentech, off-label use) [62]. These intravitreal anti-angiogenic agents used in clinical settings are based on limited activity against factors belonging to the VEGF family. This entails the inhibition of the VEGF-A activity observed in ranibizumab and bevacizumab, as well as aflibercept which has a broader spectrum of action, neutralizing, in addition to VEGF-A, other VEGF family ligands, such as VEGF-B, the placental growth factor-1 (PlGF-1), and placental growth factor-2 (PlGF-2). Pegaptanib sodium (Macugen^®^; Bausch + Lomb; Bridgewater, NJ, US)), the first VEGF-targeting agent approved by the U.S. Food and Drug Administration (FDA), is no longer used. Recently, brolucizumab (Beovu^®^; Novartis, Basel, Switzerland) [63] and faricimab (Vabysmo; Genentech/Roche, South San Francisco, CA, USA) have been approved for ophthalmic use. Brolucizumab targets the major VEGF-A isoforms [64], but, after an initial widespread adoption, its use has since been significantly limited in certain countries following several cases of severe occlusive retinal vasculitis [65]; faricimab is the latest antibody to receive approval, and it simultaneously targets VEGF-A and angiopoietin-2 (Ang II) [66]. Biosimilars have now started to enter the market in certain healthcare systems, in an attempt to make better use of resources. In terms of treatment regimens, non-monthly treatment schedules, such as the pro re nata (PRN) strategy and the treat and extend (T&E) protocol, have garnered considerable interest. In the PRN approach, the disease status is evaluated monthly, and treatment is provided if deemed necessary. In the T&E approach, treatment intervals are fixed based on the disease status observed during each visit [67]. At present, the T&E regimen has emerged as the prevailing treatment approach on a global scale, and future research directions are concentrating on even longer intervals between injections. This highlights the need for more persistent therapeutic agents and the exploration of alternative strategies to achieve prolonged efficacy.

Among the surgical methods for administering gene therapy products targeting the inner retina, intravitreal (IVT) injections are the least invasive.

The majority of currently administered intravitreal AAVs face challenges in adequately reaching the outer retina, RPE, and choroid because of the inner limiting membrane (ILM), which acts as a physical barrier between the vitreous and the retina [68]. Although the delivery of gene therapy products to the outer retina and choroid has been enhanced with the development of recombinant vectors like AAV2.GL and AAV2.NN [69,70], the utilization of ADVM-022, an intravitreal AAV-based aflibercept gene therapy, was discontinued due to dose-limiting toxicity [71]. Nevertheless, the fact that IVT injection is more immunogenic than subretinal delivery has been highlighted [72,73] and may be useful in future studies.

### 2.2. Limitations of Anti-VEGF Therapy

Although positive results are achieved in the majority of patients, approximately 25–35% of individuals with nAMD either show suboptimal responses to existing anti-VEGF treatments, experience delayed treatment failure, or require intensive and frequent IVT therapy [74,75]. Among the 35% who do not respond optimally to therapy, more than 10% experience deterioration despite treatment, and an additional 25% exhibit no signs of improvement [76,77]. For patients who achieve disease stability, discontinuing therapy could potentially have negative consequences, necessitating the continuation of treatment at consistent intervals to maintain vision. Furthermore, in certain individuals with aggressive nAMD, the continued use of anti-VEGF therapy, even after achieving stability, does not sufficiently prevent the recurrence of the disease [78,79]. The consequences of a suboptimal response and limited effectiveness over time, leading to poor vision, significantly affect the outcomes reported by the patients. Moreover, the need for repeated treatments for nAMD places a substantial burden on healthcare systems, patients, and their caregivers. Moreover, present anti-VEGF treatments are associated with certain adverse events which, although infrequent, can considerably affect eyesight. Endophthalmitis is a severe complication that arises in approximately 1 in 3500 injections [80]. Another notable potential complication of anti-VEGF therapy is the risk of intraocular inflammation, which, if severe, may lead to irreversible vision loss [81]. Furthermore, a temporary increase in intraocular pressure is frequently noticed shortly after IVT injection of all anti-VEGF agents [82]. The repeated use of anti-VEGF treatments can also be associated with unfavorable effects. For example, macular atrophy, which represents an advanced phenotype of nAMD and may result in permanent vision loss, has been documented as being potentially linked to long-term anti-VEGF use [83]. The causative relationship is still not well-defined, and it is possible that macular atrophy is simply part of the natural history of some forms of treated MNV [84,85]; subretinal fibrosis represents another advanced manifestation of nAMD linked to permanent vision impairment and can be the result of untreated nAMD itself, which complicates any potential association between sub-retinal fibrosis and long-term anti-VEGF therapy. Exploring alternative agents that provide comparable or enhanced efficacy while requiring fewer injections and maintaining a longer duration of action could potentially address many of these limitations.

### 2.3. Emerging Therapies and Need for Alternative Treatment Options

Conbercept (Lumitin; Chengdu Kang Hong Biotech, Chengdu, China) is a recently tested agent designed to bind VEGFA, VEGF-B and PlGF [86], but the PANDA-1 and PANDA-2 phase III trials for nAMD were concluded in 2021. They were halted because the desired primary outcome, specifically the non-inferiority of conbercept compared to aflibercept, was not attained (NCT03577899 and NCT03630952). OPT-302, a novel “trap” molecule, binds to VEGF-C and VEGF-D, in turn inhibiting their activation of VEGFR2 and VEGFR3 [87]. This new agent is currently being assessed in phase III trials with and without either ranibizumab (ShORe trial, NCT04757610) or aflibercept (COAST trial, NCT04757636). Researchers have also attempted to develop eye drops either as potential standalone treatments [88,89] or as an adjunct to optimize IVT therapy, but they have yet to be proven effective. Port delivery systems (PDS) are innovative devices utilizing surgically implantable reservoirs that are required to be refilled periodically and have been developed to enable the continuous administration of anti-VEGF agents directly into the eye through passive diffusion [90]. The FDA initially granted approval for a PDS containing ranibizumab as a treatment option for nAMD but the product has since been recalled and is no longer available commercially. Other treatments for nAMD, focusing on the VEGF system and alternative pathways, including heparin-binding variants of VEGF receptor 1 [91] and cells derived from induced pluripotent stem cells (iPSCs), have been under assessment [92]. These multi-targeted therapies and alternative treatment options, such as retinal gene therapy, may be the answer to the unmet needs in the treatment of nAMD [93,94]. An attractive alternative approach, in fact, involves using a single intraocular injection of a gene therapy vector that would continuously express an anti-angiogenic protein to block the pathological neovascularization in AMD. The aim of this review is to summarize the rationale and progress of preclinical and clinical trials using gene delivery strategies for the treatment of nAMD.

## 3. Gene Therapy Strategies for nAMD

### 3.1. Overview of Genes Targeted

As stated above, AMD is known to be a multifactorial disease, the development and progression of which is governed by the complex interaction of various environmental and genetic elements; aging is the primary factor, and drives the overexpression of VEGF-A in the macular microenvironment among elderly patients. Advancements in technologies, such as single-cell sequencing and genome-wide association studies (GWASs), have revealed mutations and factors that contribute to the progression of AMD. Through GWASs, specific genes, including CFH on chromosome 1 and ARMS2 and HTRA1, both residing on chromosome 10, have emerged as significant loci closely linked to advanced AMD [26,95]. The CHF variant is primarily connected to the presence of drusen, whereas the ARMS2-HTRA1 variant is correlated with the occurrence of subretinal or sub-RPE hemorrhages [96]. Although these genes are involved in the development of nAMD and may be useful predictors of treatment response, they have yet to be shown to have a significant role in its treatment. Other genes including MMP9, CETP, and TIMP3 have been linked to nAMD due to their roles in regulating the extracellular matrix remodeling [97], and the FGD6, HTRA1, and CFH genes play pivotal roles in governing oxidative stress and inflammation, which in turn regulate the advancement of angiogenesis, thereby contributing to the progression of nAMD [98].

However, the RPE hypoxia previously described promotes an over-expression of the hypoxia-inducible factor alpha (HIF-α) and VEGF-A by RPE cells, with the consequent degeneration of the RPE cells themselves and of Bruch’s membrane [99]. Anti-VEGF treatments have really shown that VEGFA/HIF-α-related genes (VEGF, VEGFR, PDGF, PEGF) can be useful treatments; this makes VEGF, VEGFR, PDGF, and PEGF the primary targets for the current gene therapy [100].

Gene therapy for nAMD faces challenges due to the complexity of the genes associated with the condition. Unlike monogenic disorders with a small gene that can fit into an AAV for the standard gene augmentation therapy, nAMD involves multiple genetic factors; the already mentioned genes contribute to disease susceptibility, making it challenging to devise a one-size-fits-all gene therapy. The diverse genetic landscape of nAMD adds a layer of complexity, requiring a nuanced approach in developing gene therapies tailored to the specific genetic factors.

### 3.2. Gene Silencing and Inhibition of VEGF Expression

Exploring gene silencing through small interfering RNA (siRNA) or microRNA (miRNA) targeting VEGF is considered as a potential approach for AMD treatment [101]. Numerous clinical trials are currently underway, focusing on the utilization of precise gene silencing methods [102,103,104]. After being introduced into cells, siRNA binds and activates the RNA-induced silencing complex, which in turn targets and degrades any cells complementary to the siRNA sequence, thereby preventing protein synthesis.

Bevasiranib, a modified naked RNA, results in the downregulation of VEGF-A by means of its intracellular transcriptional inhibitor action and possibly its TLR3-mediated activity, and may be the treatment of nAMD. A phase III human trial, which involved the intravitreal administration of siRNA bevasiranib (NCT00499590), was halted, as it was deemed unlikely to achieve its primary objective [105]. As bevasiranib may only inhibit new VEGF synthesis, without impacting existing VEGF levels, a phase III trial (NCT00499590) was also performed to assess the efficacy of the combined bevasiranib and ranibizumab therapy for nAMD treatment, but this too was unlikely to meet its primary endpoint and was terminated.

AGN211745 (formerly Sirna-027) is a chemically modified naked siRNA that has VEGFR-1 as the target gene, inducing gene silencing by binding the complementary target RNA with the lytic cytoplasmic protein complexes known as RNA-induced silencing complexes, thereby reducing the level of VEGFR-mRNA and significantly inhibiting MNV development, with the potential to treat nAMD. However, despite positive findings in the phase I/II study, a phase II trial administering Sirna-027 (NCT00395057) did not meet crucial efficacy endpoints. (NCT00363714) [106].

Despite many efforts in multiple trials exploring gene silencing, studies have never advanced beyond phase III, as gene silencing methods encounter several obstacles, including RNA instability, limited bioavailability, and the potential for non-specific targeting. These challenges, common to most drug delivery systems, significantly hamper the successful application of siRNA therapeutics in the treatment of nAMD. Additionally, while siRNA-based therapies have demonstrated theoretical advances for patients with nAMD, this approach has not shown any superiority compared to conventional anti-VEGF treatments. This is primarily because even with siRNA therapies, the requirement for repeated injections persists, as their effect is temporary (3–7 days) due to their degradation by tissue nucleases. Nonetheless, the possibility of extending these effects exists through chemical alterations or the use of viral vectors, which could help maintain the efficacy of therapies based on RNA interference.

An alternative to siRNAs involves the use of microRNAs (miRNAs) which are small (18–22 nucleotide), single-stranded, noncoding RNAs that down-regulate the gene expression post-transcriptionally [107]. Various research studies have shown that the dysregulation of miRNAs is relevant both in experimental AMD models and in AMD subjects, and may therefore potentially be associated with an increased risk of developing AMD [108,109,110]. MicroRNA mimics or anti-miRNA have the potential to be biomarkers, diagnostic tools, or targets for the control and treatment of this disease, by modulating retinal cellular function [111]. Unfortunately, the miRNAs evaluated in animal models of AMD behave differently compared to AMD patients; thus, their role in the disease remains unclear [112].

### 3.3. Gene Delivery Approaches: Viral Vector-Based and Non-Viral Delivery

To achieve successful results in gene therapy, it is essential to use a vector that ensures prolonged gene expression levels while minimizing the risks of toxicity and immune reactions. Different types of vectors have been used.

#### 3.3.1. Viral Vector-Based Delivery

Viral vectors are modified viruses commonly used in gene therapy approaches to deliver therapeutic genes or RNA-based molecules to target disease cells. They have been used as delivery vehicles to precisely transport therapeutic genetic material into the target cells within the eye and achieve a sustained therapeutic effect. In gene therapy for nAMD, vector selection is of paramount importance. For retinal gene supplementation, the optimal selection is the recombinant adeno-associated viral vector (AAV) [113,114]. Its small, single-stranded DNA genome of approximately 4.6 kilobases (kb) with organized capsid structure makes it conducive to genetic modifications [115]. AAVs are currently the most commonly used vector for retinal gene transfer in both preclinical studies and clinical trials [116]. They provide advantages like extended transgene expression, minimal risk of insertional mutagenesis, only slight inflammatory responses induced, and a low chance of germline transmission [117,118]. The most extensive AAV serotypes studied in ocular gene therapies are AAV2, AAV5 and AAV8 [119,120,121]. Gene therapy products utilizing AAV vector systems, including Glybera (alipogene tiparvovec to treat hereditary lipoprotein lipase deficiency) [122], Luxturna (voretigene neparvovec-rzyl), Zolgensma (onasemnogene abeparvovec to treat spinal muscular atrophy type 1) [123] and Hemgenix (Etranacogene dezaparvovec for the treatment of hemophilia B) [124], have received notable approvals. Among these, Luxturna, the first approved gene therapy for a genetic disease, is a recombinant AAV 2 vector containing human *RPE65* complementary DNA that enables RPE cells to produce the retinoid isomerohydrolase RPE65. After its efficacy and safety were ultimately confirmed in an open-label, randomized and controlled phase 3 trial conducted at two centers in the United States, Luxturna was authorized for gene augmentation therapy in *RPE65*-associated retinal dystrophy [125] and stands out as a retinal gene therapy designed to treat Leber congenital amaurosis (LCA) [126]. However, a subset of patients undergoing subretinal Luxturna injection developed progressive perifoveal chorioretinal atrophy following surgery. Despite that, most patients did well on visual function measures. Although the mechanism for chorioretinal atrophy is not known at this time, there are several potential factors that must be considered, alone or in combination, namely: direct toxicity of the AAV2 vector to the photoreceptors and RPE, inflammation or immune response to the vector, surgical delivery and ocular factors [127]. Further studies are necessary to determine what potential factors predispose patients to this complication and to clarify what the implications are for gene therapy in nAMD, especially in terms of a immune response.

Moreover, retroviruses and lentiviruses have been employed in various gene therapy products, such as RetinoStat^®^ (Oxford BioMedica, Oxford, UK, OXB-201) targeted for nAMD (NCT01301443) and stem cell therapy. Notably, subretinal administration of RetinoStat, a lentiviral vector expressing endostatin and angiostatin, demonstrated safety and good tolerance. Patients with severe nAMD exhibited signs of clinical improvement, including visual acuity stabilization and reduction in vascular leakage [128]. Nonetheless, retroviruses and lentiviruses carry risks such as the potential for insertional mutagenesis and germline transmission. Additionally, they might trigger more pronounced inflammatory responses compared to AAVs. An important aspect of gene therapy is the possible immune reaction towards the AAV capsid: in humans, administering AAV vectors, unlike in many animal models, triggers antigen-specific T-cell activation, posing an increased risk during the initial postoperative phase. A brief period of immunosuppression around the surgery can help regulate immune responses until the capsid antigens are eliminated from the infected cells [129]. The route of vector delivery significantly influences immunogenicity. Subretinal delivery is a favorable option for disorders primarily affecting the RPE and/or photoreceptors. Given that the majority of inherited retinal disorders (IRD) involve either or both of these cell types, the subretinal delivery emerges as the prevailing administration route in gene therapy trials targeting monogenic conditions. This method involves the creation of a retinotomy near the temporal vascular arcades, allowing the bleb to slowly spread toward the foveal region, creating a shallow elevation [68]. Despite this type of delivery method involving a temporary detachment of the retina, the existing trial data indicates that it is generally safe and has the potential to offer effective therapeutic outcomes [69,70,71].

#### 3.3.2. Non-Viral Delivery

Among the non-viral delivery techniques, the most straightforward approach is physical delivery, which involves injecting naked plasmid DNA, siRNA, mRNA or miRNA. However, this method has a limited efficacy due to the rapid degradation and minimal uptake [130]. Non-viral gene delivery through chemical techniques is attractive due to its lower potential to trigger immune responses, straightforward scalability and cost savings in production [131].

DNA nanoparticles consisting of a single molecule, compacted using polyethylene glycol (PEG)-substituted lysine peptides (CK30-PEG), have been utilized for transporting payloads of up to 20 kb in size [132,133]. These nanoparticles have demonstrated safety in diverse mouse models of retinal degeneration [134,135].

Lipid-based transfection systems have proven to be effective in delivering target genes to retinal cells in various studies. Numerous lipid-based drugs designed for eye diseases are accessible for transporting CRISPR or ribonucleoproteins for base editing [136,137]. Niosomes, consisting of cholesterol and uncharged single-chain surfactant [138,139], exhibit potential as non-viral carriers for gene delivery [140,141,142]. In ocular gene therapy, polymer-based platforms like chitosan, hyaluronic acid, polyethyleneimine (PEI), poly(amidoamine) (PAMAM), PEG, poly (lactic-glycolic acid) (PLGA) and poly(L-lysine) (PLL) have been under investigation [101,130].

### 3.4. Gene Editing Technologies and CRISPR/Cas9

Gene editing technology involves the manipulation of the target gene at the DNA or genomic level. The most common gene editing system to date uses clustered, regularly interspaced, short palindromic repeat (CRISPR) endonucleases such as Cas9, which can cut the DNA at a precise, targeted location, to either ablate or repair a destructive mutation [143]. The CRISPR/Cas9 system comprises a guide RNA targeting the gene of interest and an endonuclease that creates a site-specific double-stranded DNA “cut”, enabling precise genetic modification [144]. This allows for the lasting and accurate modification or removal of a mutation associated with a specific disease [145]. However, when addressing mutations in a single gene, CRISPR may not be effective for patients without a recognized genetic diagnosis. The CRISPR-Cas9 system has several potential advantages over other editing tools such as its simplicity of target design, ease of generating large-scale libraries and relatively low cost [146,147]. Moreover, the genome editing with CRISPR-Cas9 enables multiple editing through the engagement of multiple guide RNAs (gRNAs) [148,149]. Treatment for AMD patients can involve the use of the adeno-associated viral vector (AAV)-CRISPR tool, utilizing CjCas9 (*Campylobacter jejuni*) [150,151], and type-V CRISPR-Cas systems with LbCpf1 nucleases. AAV-delivered CjCas9 can accurately target and modify specific sites in the human or mouse genome, inducing mutations in RPE cells. In this context, CjCas9 can target the VEGFA or Hif1a gene in RPE cells, potentially reducing the size of laser-induced neovascularization. This approach may evolve into an in vivo genome editing therapy for nAMD [152]. Progress in CRISPR/Cas9 technology, including base and prime editing, holds the promise of improving the efficiency and cost-effectiveness of using CRISPR/Cas9 to treat retinal diseases such as nAMD.

However, a key challenge in the application of CRISPR/Cas9 technology remains the manufacturing and production for in vivo editing [153], and all CRISPR applications in retinal diseases including nAMD have been largely experimental; clinical trials of CRISPR for nAMD are lacking, as the field is still exploring safety and efficacy concerns.

The genomic impacts of transduction using AAV vectors encoding CRISPR-Cas nucleases are still under investigation; high levels of AAV integration (up to 47%) into Cas9-induced double-strand breaks (DSBs) are in therapeutically relevant genes in cultured murine neurons, mouse brain, muscle, and cochlea, and this should be recognized as a common outcome for applications that utilize AAV for genome editing [113]. Moreover, efficient gene delivery and editing can be achieved through the ocular delivery of mRNA packaged in lipid nanoparticles (LNPs). Subretinal injections of LNPa containing Cre mRNA in the mouse show a tdTomato signal in the RPE, enabling genome editing in the retina; in the future, this can be used to correct genetic mutations that lead to blindness [114].

## 4. Clinical Trials and Promising Gene Therapy Approaches

Clinical trials investigating gene therapy for nAMD currently adopt two strategies: the intraocular administration of modified viral vectors expressing antiangiogenic proteins, and RNA interference molecules to contrast the VEGF overexpression.

To this purpose, PEDF, endostatin, angiostatin, secreted extracellular domain of VEGFR1 and sFLT-1 have been targeted by gene therapy [154].

### 4.1. PEDF

A phase I clinical trial (ClinicalTrials.gov: NCT00109499) explored the safety of AdGVPEDF.11D in patients affected by advanced nAMD. The investigators delivered the PEDF gene via an adenoviral vector with deficient replication (by deletion of E1, E3, and E4). PEDF is an important endogenous antiangiogenic factor, and its levels are low in the presence of nAMD. Adenovirus, a double-strand DNA virus, can carry up to 37 kb for transgene delivery [155,156,157,158]. The participants received an intravitreal injection of AdGVPEDF.11D with dosages ranging from 1E6 and 1E9 particle units (PU). In 25% of cases, there were reports of mild and temporary intraocular inflammation, with no severe adverse events. Although the study was not designed to assess the therapeutic efficacy, neovascularization was observed to be stable or reduced in patients receiving 1E8 or 1E9 PU, compared to those receiving lower doses.

### 4.2. Anti-VEGF

Intravitreal and subretinal injection of FLT-1 (also known as VEGFR-1) or FLT-1 derivates have been tested on nAMD patients after encouraging results on animal models [159]. FLT-1 expression is normally upregulated by hypoxia, neutralizing VEGF-A, and thereby preventing its dimerization with membrane receptor VEGFR-2 and the consequent pro-angiogenic pathway. The intravitreal injection of AAV2-sFLT01, encoding for a fusion protein composed by sFLT-1 domain 2 and the Fc domain of IgG_1_, was tested in a phase I trial (ClinicalTrials.gov: NCT01024998, Sanofi Genzyme, Paris, France), whereas the subretinal administration of recombinant AAV (rAAV).sFLT-1, encoding the natural soluble FLT-1, was experimented on in a phase I/IIa trial (ClinicalTrials.gov: NCT01494805, Avalanche Biotechnologies).

In the first trial, the viral vector was demonstrated to be safe, not detectable systemically and not eliciting immunogenic activity. Moreover, the encoded protein was detectable within 52 weeks in 5 of the 10 patients treated with the highest dosage (2E10 vector genomes). In general, the expression was dose-related, but variable among the subjects, with 80% of non-expressers showing, at baseline, anti-AAV2 antibody titers of 1:400 or greater, indicating a considerable impact of individual characteristics in determining the response to treatment. Although the treatment was well tolerated at all dosages, it did not produce any significant anatomical (retinal thickness) and functional (BCVA) improvement [160].

The phase I/IIa trial NCT01494805 confirmed the safety and effectiveness of the subretinal injection of the rAAV.sFlt-1 vector, resulting in an increase in retinal sFLT-1 levels. Forty patients suffering from nAMD were assigned to low-dose, high-dose or control arms. A regular intravitreal injection of ranibizumab was administered when patients showed a BCVA reduction or intraretinal/subretinal fluid increase on OCT or augmented leakage on fluorescein angiography during the 36-month follow-up. The number of intravitreal treatments and changes in BCVA and retinal thickness were recorded during the 36-month follow-up. This gene therapy demonstrated safety and good tolerance; however, no notable changes were observed in the examined endpoints [161,162,163]. The induced endogenous expression of anti-VEGF has also been explored in humans, after encouraging results on animal models.

In a phase I clinical trial (ClinicalTrials.gov: NCT03748784, Adverum Biotechnologies, Redwood City, CA, USA), an AAV2-derived vector, 7m8 (AAV.7m8-aflibercept), named ADVM-22, was administered via intravitreal injection in 18 nAMD aflibercept-responder patients. Aflibercept expression, BCVA, OCT changes and the need for a rescue treatment with standard intravitreal injection of aflibercept were assessed. The BCVA maintenance and retinal thickness reduction on OCT were observed in 12 patients who received 2E11 or 6E11 doses of ADVM-22, with 10 of them (83%) not requiring rescue treatment for about 11 months [164]. The ADVM-22 was assessed in the INFINITY trial for diabetic macular edema (DME) and in the OPTIC clinical trial in patients with nAMD. The data from the studies show marked differences in the safety profile between the two patient populations, with rapid, clinically relevant decreases in intraocular pressure refractory to steroids, requiring subsequent additional treatment in the treated eye of some of the patients with DME. Although no similar clinically relevant events were observed in the OPTIC trial patients, this unexpected occurrence has disrupted further evaluation of the intervention [165].

Positive results were also obtained with the delivery of a gene encoding a soluble monoclonal portion of an anti-VEGF antibody structurally similar to ranibizumab. The safety and tolerability of this gene treatment, called RGX-314 and administered via subretinal injection, was tested in a phase I/IIa trial (ClinicalTrials.gov: NCT03066258, REGENXBIO). The 42 enrolled nAMD patients had previously been treated with anti-VEGF intravitreal injections. They were divided into 5 cohorts receiving the adeno-associated viral vector (NAV AAV8) at different doses (3E9, 1E10, 6E10, 1.6E11 and 2.5E11 genome copies [GC] per eye). The rescue treatment consisted of intravitreal anti-VEGF in the case of vision loss of 5 or more ETDRS letters; persistent, increased or new intra/subretinal fluid on OCT; or the appearance of new macular hemorrhage. The aqueous levels of the encoded protein were observed to increase in a dose-dependent manner in the five subgroups, with the RGX-314 protein reaching 260.5 ng/mL in 1 year in the 6E10 cohort (six patients). In the same sub-group at 24 months, BCVA was improved by 14 ETDRS letters, and the central retinal thickness remained stable at the baseline. BCVA remained stable at 2 years, with changes within one ETDRS line in cohort 2, 4, and 5. The rescue treatment after 2 years was necessary in all cohorts, with a lower mean number in the higher dose cohorts (2.8, 4.4 and 2 ranibizumab injections in cohorts 3, 4 and 5, respectively), whereas the first two cohorts received a higher number of rescue injections (10.3 and 9.3 in cohort 1 and 2, respectively). RGX-314 showed a good tolerability; overall, the intervention demonstrated no severe adverse events at the lower doses; 2 participants in the highest dose group developed retinal pigmentary changes that resulted in vision loss. As a consequence, the protocol was amended [166]. These results encouraged several more studies on RGX-314 safety and efficacy on nAMD patients: a phase II trial (ClinicalTrials.gov: NCT04832724) comparing the effects of two different doses in two subretinal formulations, the clinical and the eventual commercial formulations, a phase II trial (ClinicalTrials.gov: NCT04514653) comparing 3 different doses of RGX-314 with ranibizumab, a randomized combination of a fixed dose of RGX-314 with either topical or local steroid formulations post-treatment, and a 5-year follow-up trial with a sub-study on the affected fellow-eye (ClinicalTrials.gov: NCT03999801). Unfortunately, no conclusive data from these trials are currently available [167].

### 4.3. Endostatins and Angiostatins

The subretinal injection of viral vectors encoding endostatin and angiostatin, which are endogenous inhibitors of angiogenesis, showed good preclinical results on mice with laser-induced neovascularization [168,169]. These results prompted a phase I clinical study (ClinicalTrials.gov: NCT01301443, Oxford Biomedica) on subretinal treatment with the non-replicating bicistronic EIAV vector encoding both endostatin and angiostatin (RetinoStat) on humans with advanced nAMD. The trial enrolled 21 patients that were divided into three cohorts receiving a different treatment dose (4E4, 2.4E5 and 8E5 transduction units [TU]). The gene therapy was safe, well tolerated and generated a sustained expression of angiostatin and endostatin, which was detected in aqueous humor samples of eight patients for up to 2.5 years and in 2 patients for more than 4 years. Unfortunately, despite a documented reduction of fluorescein leakage, the treatment produced no functional improvement [170].

### 4.4. Complement Cascade Inhibition

The complement cascade activation with membrane attack complex (MAC) accumulation has been observed to be upregulated in AMD patients, with consequent RPE cell damage. This process is thought to play an important pathogenetic role in both atrophic and nAMD [171]. CD59 is a membrane that prevents MAC formation on the cell membrane in the final phases of the complement cascade leading to cell lysis. Therefore, a soluble form of this molecule has been studied for gene therapy applications in dry AMD and nAMD. For the latter, a phase I trial (NCT03585556) adopting the intravitreal injection of AAVCAGsCD59, a viral vector encoding for soluble CD59 was initiated, but the results have yet to be made available.

### 4.5. RNA Interference

Another gene-based therapeutic strategy to reduce the expression of VEGF and its receptors is gene silencing with siRNAs. As previously mentioned, these artificial RNA strands are capable of forming complexes with complementary mRNA, selectively silencing their expression after transcription. Preclinical successes on nAMD models led to clinical trials adopting bevasiranib, a 21-nt RNA silencing the VEGF encoding mRNA, and ANG 211745, a 21-nt RNA that silences the mRNA encoding for FLT-1, also known as VEGFR1.

Bevasiranib was the first siRNA approved for IVT use in clinical trials on nAMD patients. This treatment was proved safe in a phase I trial (ClinicalTrials.gov: NCT00722384, OPKO Health) adopting five dosing regimens (0.1, 0.33, 1, 1.5 and 3 mg). Since bevasiranib inhibits VEGF synthesis, but does not affect the preexisting VEGF levels, and its efficacy as a monotherapy was demonstrated insufficient in a phase II trial (ClinicalTrials.gov: NCT00259753), resulting in BCVA loss and neovascular lesion enlargement, this gene therapy was subsequently associated with intravitreal ranibizumab in patients affected by nAMD in a phase III trial (ClinicalTrials.gov: NCT00499590). Despite its promising rationale, the trial did not meet its primary endpoint and was terminated.

Another phase III trial testing the efficacy and safety of the combined bevasirinab-ranibizumab therapy was aborted even before the enrollment started due to concerns regarding its Toll-like receptor (TLR) action, which was detected in murine models, and observed to induce RPE cell apoptosis [105].

The first phase I trial (ClinicalTrials.gov: NCT00363714, Allergan, Dublin, Ireland) assessing another intravitreal si RNA (ANG 211745) safety in nAMD patients showed good results, but the following phase II trial failed to reach its therapeutic targets [106]. Further concerns emerged on the TLR3 pathway activation; thus, more specific gene treatments were developed to overcome this limitation. In a phase I trial, the intravitreal injection of PF-045236 (ClinicalTrials.gov: NCT00725685), a 19-nt siRNA silencing the hypoxia-induced gene RTP801, was tested on patients with MNV or DME and was demonstrated to be safe and well tolerated [172]. In the subsequent phase II MONET trial (ClinicalTrials.gov: NCT00713518), this gene therapy showed no superiority in improving BCVA when compared with ranibizumab, but the two treatments combined showed synergetic efficacy [173].

The need to frequently combine treatment regimes in order to obtain the best outcome for the patient highlights the complexity of nAMD pathogenesis and, consequently, the need for a multifactorial therapeutic approach. To this purpose, a single gene therapy that regulates the expression of different proangiogenic molecules simultaneously would represent the ideal solution; preclinical studies by Askou et al. evaluated multigenic lentiviral vectors in human cells and in mouse retina that encode for both PEDF and anti-VEGF miRNA [174].

Several other clinical trials are currently recruiting, but the results are still awaited. These trials are included in Table 1 but have not been discussed in this review, as conclusive data have yet to be published.

## 5. Conclusions and Future Perspective

AMD is a complex disease characterized by a variety of genetic and molecular factors contributing to its pathogenesis and development. The approach to date for nAMD management is VEGF-A-based antiangiogenics; currently, the developed therapeutics are anti-VEGF antibodies or recombinant fusion proteins. However, the monthly repetitive intravitreal injection of these agents only achieves a limited control of nAMD, and its progression.

Gene therapy is a rapidly evolving field and is radically different from previously available forms of treatment. It has both advantages and potential disadvantages, but it could reduce the treatment burden by providing sustained and long-lasting therapeutic effects; when a therapeutic gene is successfully integrated into patient cells, it can continuously produce the desired protein, which in the case of nAMD most likely results in the down-regulation of VEGF. The need for a reduced frequency of treatment clearly has advantages for the patient, with significant improvement in the quality of life and preservation of vision and a reduction in the socio-economic burden associated with sight loss. Viral vector gene therapy appears to be the most promising option in nAMD, but the multifactorial character of the disease implies that gene therapy will require considerable effort to realize desirable therapeutic outcomes.

Furthermore, gene therapy raises several concerns. The potential off-target effects have already been mentioned; however, the main safety concern is ensuring precise control over gene expression; once a gene is introduced or modified in a cell, it cannot be easily switched off. This is of particular concern if the therapeutic gene has the potential to produce a protein that may be harmful if overexpressed. Furthermore, once a gene is modified, there is limited control over its expression; this is important, as the level of function may not be adjusted according to changing patient needs [175,176].

Moreover, the selection of promoters plays a crucial role in gene therapy for retinal diseases. Promoters are DNA sequences that control the initiation of gene expression, determining when and where a therapeutic gene is activated. In retinal gene therapy, the choice of promoter influences the specificity, strength and duration of gene expression within the target cells. Different retinal diseases may require distinct promoter characteristics. Precision in promoter selection helps avoid off-target effects and enhances the therapeutic gene’s therapeutic efficacy. Additionally, the durability of gene expression is a critical consideration. Some diseases may benefit from sustained expression over an extended period, necessitating the use of promoters that support long-term gene activity. Overall, the strategic choice of promoters in nAMD gene therapy is pivotal for optimizing treatment outcomes, tailoring expression patterns to specific cell types, and achieving the desired therapeutic effects while minimizing unintended consequences [177].

The number of studies reported in the literature is vast; this review does provide an exhaustive analysis but represents the issues the authors considered pertinent regarding gene therapy for nAMD. While gene therapy approaches, including those involving AAV vectors and CRISPR-Cas nucleases, are actively being explored, their widespread clinical application for nAMD is still in the investigational stage.

The results of the clinical trials described in this review may lay the foundations to revolutionize treatment plans for nAMD in the future. Challenges and areas of controversy persist, but progress has been made to optimize the dosage of these drugs, the routes of administration, post-injection management and long-term benefits. Future gene therapy studies need to live up to several key expectations to advance the field effectively. Future studies should focus on enhancing the precision and specificity of gene therapies. This involves developing technologies that can accurately target specific cells or tissues, minimizing off-target effects. The sustainability of gene therapies is crucial; ensuring that the therapeutic effects persist over the long term is essential for the success of these treatments, particularly in chronic conditions like nAMD. Continued efforts must be made to improve the safety profiles of gene therapies. This includes minimizing adverse reactions, immune responses, and other potential risks associated with the delivery of therapeutic genes. Moreover, gene therapy approaches can be combined with other treatment modalities, such as anti-VEGF therapies, to potentially enhance the overall effectiveness of treatment. While gene therapy for nAMD is still in its early stages, ongoing research and advancements in gene delivery methods, vector development, and gene editing technologies hold promise for improving its efficacy and safety profile and advancing the potential of clinical applications and patient benefit.

## Figures and Tables

**Table 1:** Clinical trials investigating gene therapy for nAMD.

Trial ID	Development	Tested Drug	Route of Administration	Mechanism	Results	
NCT00109499	Phase I	AdGVPEDF.11D	Intravitreal injection	Induction of PEDF expression	Safe, MNV size stable or reduced with dose 1E8 or 1E9 PU	
NCT01024998	Phase I	AAV2-sFLT01	Intravitreal injection	Induction of gene AAV2-sFLT01 encoding for an anti-angiogenic fusion protein formed by FLT-1 and IgG_1_ Fc domain that neutralizes VEGF-A before it binds its receptor	Safe, good protein expression levels, but without significant anatomo-functional results	
NCT01494805	Phase I/IIa	AAV (rAAV).sFLT-1	Subretinal injection	Induction of gene encoding the natural anti-angiogenic protein FLT-1 that neutralizes VEGF-A before it binds its receptor	Safe, no significant anatomo-functional results	
NCT03748784	Phase I	AAV.7m8-aflibercept	Intravitreal injection	Induction of endogenous aflibercept expression in confirmed exogenous aflibercept-responding patients	BCVA and retinal thickness maintenance in 12 patients out of 18 (10 of them not requiring rescue treatment for about 11 months)	
NCT03066258	Phase I/IIa	RGX-314 (5 cohorts with different doses)	Subretinal injection	Induction of endogenous anti-VEGF protein (similar to ranibizumab) expression in confirmed ranibizumab-responding patients Induction of endogenous anti-VEGF protein (similar to ranibizumab) expression in confirmed ranibizumab-responding patients	Safe, good efficacy with functional and anatomical stabilization or improvement and less rescue treatments in patients treated with higher doses	
NCT04832724	Phase II	RGX-314 (clinical vs. eventual commercial formulation)			Recruiting, data not available	
NCT04514653	Phase II	RGX-314 vs. ranibizumab				
NCT03999801	Phase II	RGX-314 vs. ranibizumab and RGX-314 + local vs. RGX-314 + topical steroids				
NCT01301443	Phase I	RetinoStat	Subretinal injection	Induction of supplemental endogenous endostatin and angiostatin expression	Safe. Non-significant effectiveness	
NCT03585556	Phase I	AAVCAGsCD59	Intravitreal injection	Induction of soluble CD59 expression to prevent MAC formation and cellular damage and apoptosis	Data not available	
NCT00722384	Phase I	Bevasiranib	Intravitreal injection	Post-transcription silencing of VEGF mRNA	Safe	
NCT00259753	Phase II	Bevasiranib			Vision loss and MNV extension	
NCT00499590	Phase III	Bevasiranib combined with intravitreal ranibizumab			Terminated due to missed primary endpoints	
NCT00363714	Phase I	AGN 211745	Intravitreal injection	Post-transcription silencing of FLT-1 (VEGFR-2) mRNA	Safe	
NCT00725685	Phase I	PF-04523655	Intravitreal injection	Post-transcription silencing of hypoxia-induced gene RTP801	Safe	
NCT00713518	Phase II	PF-04523655 versus Ranibizumab	Intravitreal injection	A 19-nucleotide methylated double stranded siRNA targeting the RTP801 gene	Not significantly more effective than ranbizumab, but synergetic with it in improving BCVA	
NCT05657301	Phase I	KH631	Subretinal injection	Adeno-associated virus 8 vector that encodes a human VEGF receptor fusion protein	Recruiting, no results posted	
**NCT05672121**	Phase I/II	KH631	Subretinal injection	Adeno-associated virus 8 vector that encodes a human VEGF receptor fusion protein	Recruiting, no results posted	
NCT05536973	Phase II	ADVM-022 (AAV.7m8-aflibercept)	Intravitreal injection	Induction of endogenous aflibercept expression in confirmed exogenous aflibercept-responding patients	Recruiting, no results posted	
NCT05197270	Phase I/II	4D-150	Intravitreal injection	Dual transgene payload, expressing aflibercept and an anti-VEGF-C RNAi	Recruiting, no results posted	
NCT06031727	Phase I	HG202	Not specified	Knockdown of Vascular Endothelial Growth Factor A	Recruiting, no results posted	
NCT05903794	Phase I	EXG102-031	Not specified	Expressing a fusion protein that is able to bind all subtypes of VEGF as well as the angiopoietin 2	Recruiting, no results posted	
NCT05099094	Phase I	IDLV	Intravitreal/intracameral/subretinal	IDLV vector is engineered to carry the VEGFA antibody gene	Recruiting, no results posted	
NCT05407636	Phase III	RGX-314	Subretinal/suprachoroidal	Induction of endogenous anti-VEGF protein	Recruiting, no results posted	
NCT04704921	Phase IIb/III	RGX-314	Subretinal/suprachoroidal	Induction of endogenous anti-VEGF protein	Recruiting, no results posted	

PEDF = pigment epithelium derived factor; MNV = macular neovascularization; VEGF = vascular endothelial growth factor; PU = particle units; VEGFR = vascular endothelial growth factor receptor.
